# Real‐world data on STRIDE‐II treatment targets in a pediatric cohort with inflammatory bowel disease

**DOI:** 10.1002/jpn3.70345

**Published:** 2026-01-18

**Authors:** Marie‐Luise Frank, Thu Giang Le Thi, Ina Schacker, Lena Bragagna, Milena Geist, Simon Buehler, Victoria Riedl, Sibylle Koletzko, Tobias Schwerd, Hannes Hoelz

**Affiliations:** ^1^ Department of Pediatrics Dr. von Hauner Children's Hospital, University Hospital Munich Germany; ^2^ Department of Pediatrics, Child Health Foundation Dr. von Hauner Children's Hospital, LMU University Hospital Munich Germany; ^3^ Department of Pediatrics, Gastroenterology and Nutrition School of Medicine Collegium Medicum University of Warmia and Mazury Olsztyn Poland

**Keywords:** Crohn's disease, predictors of poor outcome, treatment stratification, treat‐to‐target, ulcerative colitis

## Abstract

**Objectives:**

STRIDE (selecting therapeutic targets in inflammatory bowel disease) established evidence‐based targets for treat‐to‐target strategies in inflammatory bowel disease (IBD). STRIDE‐II designates clinical remission, C‐reactive protein (CRP) normalization, and fecal calprotectin (FC) reduction as short‐ to intermediate‐term targets, and mucosal healing as a long‐term target. This study evaluated STRIDE‐II application and disease control in a real‐world pediatric cohort.

**Methods:**

We retrospectively analyzed newly diagnosed pediatric IBD patients (ages 3–18 years) treated according to guidelines between June 2017 and January 2023. Time‐to‐reach STRIDE‐II targets and time‐to‐first flare were assessed over 52 weeks using disease activity indices, inflammatory biomarkers (CRP, FC), and endoscopy.

**Results:**

Seventy‐four patients were included (37 Crohn's disease [CD], 37 ulcerative colitis [UC]). All CD patients received primary maintenance therapy with immunomodulators or biologics, versus 51% UC patients. Clinical remission and CRP normalization occurred within 5–10 weeks; FC normalization within 12–19 weeks. By 6 months, combined targets (clinical remission plus CRP and FC normalization) were achieved by 54% of CD patients and 43% of UC patients. Clinical relapse after remission occurred more frequently in UC than in CD (67% vs. 43%, *p* = 0.0334). Follow‐up endoscopy at a median of 41 weeks (CD) and 52 weeks (UC) showed endoscopic healing in 7/18 (39%) CD and 13/22 (59%) UC patients.

**Conclusions:**

Most pediatric IBD patients achieved clinical remission and CRP normalization within STRIDE‐II timeframes, whereas FC normalization occurred later, and relapses—particularly in UC—remained common. The notable proportion of patients with suboptimal disease control underscores the need for continuous monitoring in pediatric IBD.

## Abstract (highlights)

**What is Known:**

- Selecting therapeutic targets in inflammatory bowel disease consensus II (STRIDE‐II) is based on the treat‐to‐target approach with short, intermediate, and long‐term targets.
- The algorithm of STRIDE‐II is based on a risk assessment, frequent monitoring, and treatment adaptation.

**What is New:**

- This study provides real‐world data on STRIDE‐II treatment targets in a pediatric cohort.
- Clinical remission and normalization in biomarkers were achieved earlier in ulcerative colitis (UC) than in Crohn's disease.
- Relapse within the first year postdiagnosis occurred earlier and more frequently in UC.

## Graphical abstract

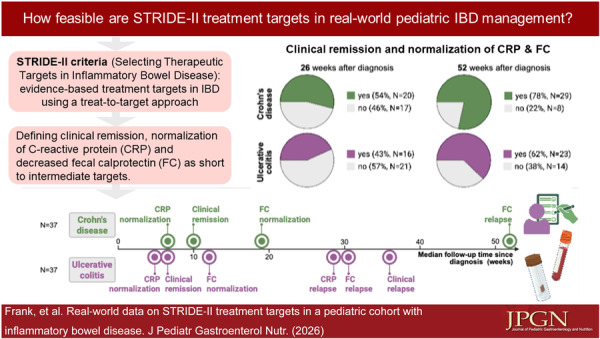

## 1 INTRODUCTION

Crohn's disease (CD) and ulcerative colitis (UC) are chronic idiopathic inflammatory bowel diseases (IBD) characterized by chronic inflammation with a relapsing disease course. Approximately 25% of patients present before age 18.^1^ Pediatric‐onset IBD (pIBD) typically follows a more severe course, frequently requiring intensified immunosuppressive therapy, suggesting a more aggressive phenotype.^1^, ^2^ Incidence and prevalence of pIBD are rising^3^, ^4^ with European prevalence ranging from 31.0 to 75.0 per 100.000 people, with Germany among the highest.^4^ Although clinical disease activity correlates well with endoscopic activity in UC,^5^ symptom‐based step‐up treatment alone has been insufficient to alter the progression of IBD. To produce better outcomes, treatment in a specific window of opportunity, before bowel damage occurs, is recommended.^6^, ^7^ Therefore, early, target‐driven therapy applying objective measures of disease activity is essential to prevent tissue damage and long‐term disability.

The treat‐to‐target strategy involves risk stratification, regular monitoring, and dynamic treatment adaptation to predefined goals .^7^ Treatment targets have evolved from symptom control to “deep remission,” including clinical and mucosal healing, with early mucosal healing linked to improved long‐term outcomes.^8^, ^9^, ^10^ In 2015, the International Organization for the Study of IBD (IOIBD) published STRIDE (Selecting Therapeutic Targets in Inflammatory Bowel Disease), defining clinical remission and mucosal healing as long‐term targets.^11^ The 2020 STRIDE‐II update includes pediatric‐specific guidance and introduces a tiered framework of short‐, intermediate‐, and long‐term targets.^12^ These include C‐reactive protein (CRP) normalization and fecal calprotectin (FC) reduction as early indicators of response. Monitoring is recommended every 3 months if active disease persists, then every 6–12 months. Endoscopy should be performed at 3–6 months in UC and 6–9 months in CD after initiating therapy.

We analyzed STRIDE‐II target achievement in a real‐world cohort of pediatric IBD patients during the first year postdiagnosis.

## 2 METHODS

### 2.1 Ethics statement

Written informed consent for analysis of clinical data was obtained from all patients or guardians at diagnosis. The study was approved by the Ethics Committee of the Ludwig‐Maximilians‐University (LMU) Munich (approval no. 25‐0523).

### 2.2 Participants

This retrospective study included children and adolescents aged 3–18 years with newly diagnosed IBD, treated at the Department of Pediatric Gastroenterology, Hepatology and Nutrition, Dr. von Hauner Children's Hospital, LMU Munich, between June 2015 and January 2023. Patients with monogenetic IBD or prior IBD‐related surgery were excluded. Diagnosis followed the revised Porto criteria. Clinical data and trajectories of FC, CRP, and erythrocyte sedimentation rate (ESR), along with endoscopic and histologic healing, were analyzed. Treatment followed European Crohn's and Colitis Organisation (ECCO)/ European Society for Pediatric Gastroenterology, Hepatology, and Nutrition (ESPGHAN) guidelines.^13^, ^14^, ^15^, ^16^

In CD, therapy was risk‐adapted as suggested by van Rheenen et al.^16^ Induction in luminal CD typically consisted of exclusive enteral nutrition (EEN) for 6–8 weeks; systemic steroids were added for extraintestinal manifestations. UC patients received steroids and 5‐aminosalicylates. Maintenance therapy included methotrexate, azathioprine, or anti‐tumor necrosis factor (TNF) in CD, and 5‐aminosalicylates ± azathioprine in UC. Anti‐TNF was initiated in UC after failure of conventional therapy.

### 2.3 Data collection

At diagnosis, patient demographics, disease characteristics, and details of the diagnostic work‐up (baseline laboratory values, disease activity indices, endoscopic assessment, and histological features) were recorded. Clinical disease activity was assessed using the mathematically weighted Paediatric Crohn's Disease Activity Index (wPCDAI) and Paediatric Ulcerative Colitis Activity Index (PUCAI). FC was measured using a Particel Enhanced Turbidimetric Immunoassay (PETIA, fCAL™ turbo, BÜHLMANN Laboratories AG). FC, CRP, and ESR were determined in a routine diagnostic laboratory. Height, weight, and BMI *z*‐scores were recorded at baseline and during follow‐up at intervals as close to 12 months as possible. Endoscopic findings were assessed for up to 2 years, as follow‐up endoscopies in some patients occurred more than 1 year after diagnosis. Clinical and endoscopic scores were calculated retrospectively. Patients with missing or incomplete data required for specific analyses (e.g., missing baseline values) were excluded.

### 2.4 Definitions

According to STRIDE‐II, clinical remission was defined as wPCDAI < 12.5 (CD) and PUCAI < 10 (UC).^17^ Disease activity was categorized as mild, moderate, or severe based on score thresholds (wPCDAI: 12.5–40/42.5–57.5/ > 57.5; PUCAI: 10–34/35–64/65–85). Biomarker normalization was defined as FC < 150 mg/l (CD) or <125 mg/l (UC), CRP < 0.5 mg/dL, and ESR < 20 mm/h. Time to normalization was the interval from diagnosis to the first value below threshold. Relapse was defined as a subsequent increase above the respective biomarker threshold.

Growth status and linear growth impairment were assessed according to the Paris classification (G0: normal; G1: impaired).^18^ G1 was defined by at least one criterion: (A) height *z*‐score at diagnosis lower than expected per WHO standards (≤−2 SD^19^, ^20^); (B) a difference >1.0 between observed and pre‐illness height *z*‐scores; or (C) a reduction in height *z*‐score ≥0.75 since diagnosis. Height‐, weight‐, and BMI‐for‐age *z*‐scores were calculated at baseline and follow‐up using CDC (Centers for Disease Control and Prevention) methodology (available at https://www.cdc.gov/growth-chart-training/hcp/computer-programs/sas.html; accessed on 11.10.2025).

Endoscopic remission was defined as simple endoscopic score for Crohn's disease (SES‐CD) < 3 or absence of ulcerations (CD) and Mayo endoscopic subscore (MES) = 0 (UC); histological remission as absence of inflammatory activity. Follow‐up endoscopies were evaluated if conducted within 24 months.

Clinical remission and CRP/ESR normalization were regarded as short‐term targets, FC normalization an intermediate target, and endoscopic healing a long‐term goal, based on STRIDE‐II timeframes.^17^ Suboptimal disease control (SDC) was assessed using STRIDE‐II–based “red‐flag” criteria adapted from the IBD‐PODCAST study,^21^ focusing on non‐achievement of clinical remission, FC and CRP normalization, and endoscopic healing at 26 weeks. As nearly all patients began therapy on the day of diagnosis, this was defined as treatment initiation.

### 2.5 Handling of missing data

No imputation was performed; analyses were based on complete cases. Missingness was primarily due to incomplete documentation in retrospective records, and imputation was avoided to prevent bias in estimating STRIDE‐II treatment‐target attainment and disease control in this pediatric cohort.

### 2.6 Statistical analyses

Descriptive statistics summarized patient characteristics. Distribution of continuous variables was assessed with the Shapiro–Wilk test, and *p*‐values were obtained using the Wilcoxon rank‐sum test. In time‐to‐target analyses, Gray's test compared cumulative incidence functions. Continuous variables are presented as medians with interquartile ranges, categorical variables as frequencies and percentages. A Sankey diagram visualized transitions in biomarker normalization, relapse, and disease activity from diagnosis (month 0–1) through months 3, 6, 9, and 12. For missing monthly biomarker data, the highest value within the respective quarter was used. The cumulative incidence function estimated time to first biomarker normalization or endoscopic improvement within 52 weeks, while Kaplan–Meier analysis assessed time from initial normalization to first relapse. Median times and 95% confidence intervals (CIs) were reported when applicable. Analyses were performed using SAS Enterprise Guide 8.1 (SAS Institute) and Prism 10.2.1 (GraphPad Software).

## 3 RESULTS

### 3.1 Patient characteristics

We studied disease trajectories of 74 patients, including 37 patients with CD and 37 patients with UC. Table 1 summarizes demographic and clinical characteristics of CD and UC patients, including Paris classification, extraintestinal manifestations, disease activity scores, and laboratory markers (FC and CRP) at diagnosis. Median age at diagnosis was 13 years (CD: 13.9; UC: 12.8), with ~40% female in both groups. All patients with available FC had elevated levels >150 mg/L at diagnosis. Median follow‐up was 16.0 months (CD) and 17.5 months (UC).

**Table 1:** Baseline characteristics, therapy, remission, and relapse rates during follow‐up.

Factors *n* (%) or median (IQR)	Patients with CD, N_CD_ = 37		Patients with UC, N_UC_ = 37		*p* value
Female	15 (41%)		16 (43%)		0.814^a^
Age at diagnosis (years)	13.9 (11.3, 15.2)		12.8 (9.0, 14.1)		0.165^b^
Newly diagnosed	25 (68%)		27 (73%)		0.611^a^
Paris classification (N_CD_ = 37, N_UC_ = 37)	CD location		UC extent		n.a.
	Ileal (L1)	10 (27%)	Ulcerative proctitis (E1)	1 (3%)	
	Colonic (L2)	8 (22%)	Left‐sided UC (E2)	2 (5%)	
	Ileocolonic (L3)	21 (57%)	Extensive (E3)	7 (19%)	
	Upper GI (L0)	5 (14%)	Pancolitis (E4)	27 (73%)	
	Upper GI (L4a)	25 (68%)	UC/IBD‐u severity		
	Upper GI (L4b)	2 (5%)	Never severe (S0)	28 (76%)	
	Upper GI (L4ab)	4 (11%)	Ever severe (S1)	9 (24%)	
	CD behavior		n.a		
	Nonstricturing, nonpenetrating (B1)	30 (81%)			
	Stricturing (B2)	2 (5%)			
	Penetrating (B3)	4 (11%)			
	Both penetrating and stricturing disease (B2B3)	1 (3%)			
	Perianal CD (*p*)	9 (24%)			
	CD growth				
	No growth delay (G0)	28 (76%)			
	Growth delay (G1)	2 (5%)			
Endoscopic scores (N_CD_ = 15, N_UC_ = 16)	Simple endoscopic score (SES‐CD)	11 (6, 16)	MES	2 (2, 2)	
	SES‐CD categories, *N* = 15		MES categories, *N* = 16		
	SES‐CD < 3	1 (7%)	MES = 0	0 (0%)	
	SES‐CD ≥ 3	14 (93%)	MES = 1	3 (19%)	
			MES = 2	11 (69%)	
			MES = 3	2 (12%)	
Complications and extraintestinal manifestations^a^					
Extraintestinal manifestations	13 (35%)		6 (16%)		0.062^a^
Arthritis manifestations	4 (11%)		2 (5%)		0.394^a^
Eye manifestations	1 (3%)		1 (3%)		1.000^a^
Skin manifestations	10 (27%)		4 (11%)		0.075^a^
Comorbidity	4 (11%)		4 (11%)		1.000^a^
Autoimmune disease	3 (8%)		4 (11%)		0.691^a^
Previous surgery	5 (14%)		0		0.021^a^
Fistula abscess surgery	5 (14%)		0		0.021^a^
Disease activity	wPCDAI	45 (35, 65)	PUCAI	45 (30, 60)	n.a.
	Disease activity categories^b^		Disease activity categories^b^		0.035^a^
	Mild	15 (40%)	Mild	12 (32%)	
	Moderate	11 (30%)	Moderate	21 (57%)	
	Severe	11 (30%)	Severe	4 (11%)	
Laboratory data (measurements performed at diagnosis ± 3 weeks)					
FC (mg/L), N_CD_ = 24, N_UC_ = 28	1848.5 (987.5, 4855)		1967.5 (1400, 3912.5)		0.869^b^
FC ≥ 250 mg/L, N_CD_ = 24, N_UC_ = 28	24 (100%)		27 (96%)		0.350^a^
FC ≥ 150 mg/L, N_CD_ = 24, N_UC_ = 28	24 (100%)		28 (100%)		n.a.
CRP (mg/dL), N_CD_ = 37, N_UC_ = 37	3.0 (0.8, 10.9)		3.0 (0.7, 12.8)		0.840^b^
CRP ≥ 0.5 mg/dL, N_CD_ = 37, N_UC_ = 37	32 (86%)		28 (76%)		0.235^a^
ESR (mm/h), N_CD_ = 36, N_UC_ = 31	38.5 (23, 53)		26 (9, 46)		0.086^b^
ESR ≥ 20 mm/h, N_CD_ = 36, N_UC_ = 31	28 (78%)		17 (55%)		0.046^a^
Albumin (g/L), N_CD_ = 32, N_UC_ = 23	4.1 (3.6, 4.5)		4.2 (3.6, 4.5)		0.778^b^
Albumin < 3.5 g/L, N_CD_ = 32, N_UC_ = 23	5 (16%)		4 (17%)		0.861^a^
Hb, N_CD_ = 28, N_UC_ = 22	11.4 (10.8, 12.3)		11.8 (9.1, 13.4)		0.992^b^
Therapy					
Initial therapy					<0.001^a^
EEN	29 (78%)		0		
EEN + steroids (for EIM)	3 (8%)		0		
Steroids	1 (3%)		1 (3%)		
5‐ASA	1 (3%)		11 (30%)		
5‐ASA + steroids	2 (5%)		25 (67%)		
Anti‐TNF	1 (3%)		0		
Time from diagnosis to initial therapy (weeks)	0 (0, 2)		1 (0, 2)		0.453^b^
Initial therapy duration (weeks)	7 (5, 9)		12 (12, 24)		<0.001^b^
Maintenance therapy					<0.001^a^
MTX	7 (19%)		0		
AZA	2 (5%)		5 (14%)		
Anti‐TNF	15 (41%)		9 (24%)		
Anti‐TNF + MTX	12 (32%)		2 (5%)		
Anti‐TNF + AZA	1 (3%)		3 (8%)		
5‐ASA	0		18 (49%)		
Time from diagnosis to maintenance therapy (weeks)	6 (3, 11)		18 (12, 36)		<0.001^b^
Normalization^c^ and first relapse evaluated with FC^d^					
First normalization of FC^c^, N_CD_ = 37, N_UC_ = 37	34 (92%)		32 (86%)		0.454^a^
Median time (weeks) to normalization of FC (95% CI)^e^ N_CD_ = 37, N_UC_ = 37	19 (15–30)		12 (9–31)		0.553^c^
First relapse after normalization^d^, N_CD_ = 34, N_UC_ = 32	15 (44%)		24 (75%)		0.011^a^
Median time (weeks) to first relapse (95% CI)^e^ N_CD_ = 34, N_UC_ = 32	52 (41–52)		30 (22–43)		0.0099^d^
First follow‐up endoscopy within 2 years of the initial endoscopy (104 ± 8 weeks)					
Follow‐up endoscopy performed	21 (57%)		24 (65%)		0.475^a^
Time from diagnosis to endoscopy (weeks)	41 (33‐54)		52 (34‐71)		0.600^b^
SES‐CD					n.a.
SES‐CD at follow‐up, N_CD_ = 17	3 (0, 6)		n.a		
SES‐CD < 3 at follow‐up, N_CD_ = 17	7 (41%)				
∆ SES‐CD, N_CD_ = 15	−9 (−11, −2)				
Reduction of SES‐CD ≥ 50%, N_CD_ = 15	10 (67%)				
MES, N_UC_ = 22					n.a.
MES at follow‐up, N_UC_ = 22	n.a		1 (0, 2)		
MES = 0 at follow‐up, N_UC_ = 22			6 (27%)		
∆ MES, N_UC_ = 16			−1 (−2, 0)		
Reduction of MES ≥ 1, N_UC_ = 16			11 (69%)		
Histological mucosal healing, N_CD_ = 21, N_UC_ = 22	9 (43%)		4 (18%)		0.104^a^

Abbreviations: 5‐ASA, 5‐aminosalicylic acid; AZA, azathioprine; CD, Crohn's disease; CI, confidence interval; CRP, C‐reactive protein; EEN, exclusive enteral nutrition; EIM, extraintestinal manifestation; ESR, erythrocyte sedimentation rate; FC, fecal calprotectin; GI, gastrointestinal; Hb, haemoglobin; IBD, inflammatory bowel disease; IBD‐u, Inflammatory bowel disease‐unclassified; IQR, interquartile range (25th and 75th percentile); MES, Mayo endoscopic subscore; MTX, methotrexate; PUCAI, Pediatric Ulcerative Colitis Activity Index; SES‐CD, Simple Endoscopic Score; TNF, tumor necrosis factor; UC, ulcerative colitis; wPCDAI, weighted Pediatric Crohn's Disease Activity Index.

^a^ Chi‐Square *p*‐value.

^b^ Wilcoxon rank sum *p*‐value.

^c^ Gray's test.

^d^ Logrank‐test.

^e^ Kaplan‐Meier method.

### 3.2 Therapy and disease course in the first year postdiagnosis

Induction and maintenance therapies are shown in Table 1. In line with current guidelines, most CD patients received EEN monotherapy for induction (29/37 [78%]), whereas most UC patients received 5‐aminosalicylic acid (5‐ASA) and steroids (25/37 [68%]). All CD patients received maintenance therapy with an immunomodulator (MTX or azathioprine [AZA]), anti‐TNF, or both. In UC, 19/37 (51%) received immunosuppressive or biologic maintenance, while 18/37 (49%) were treated with 5‐ASA alone. Median intervals between induction and maintenance therapy were 7 weeks (IQR 5–9) in CD and 12 weeks (IQR 12–24) in UC. All patients underwent wPCDAI or PUCAI scoring and FC, CRP, and ESR monitoring within 52 weeks of diagnosis. Median measurement frequencies are listed in Table S1.

### 3.3 Clinical remission and first relapse

**Figure 1:**
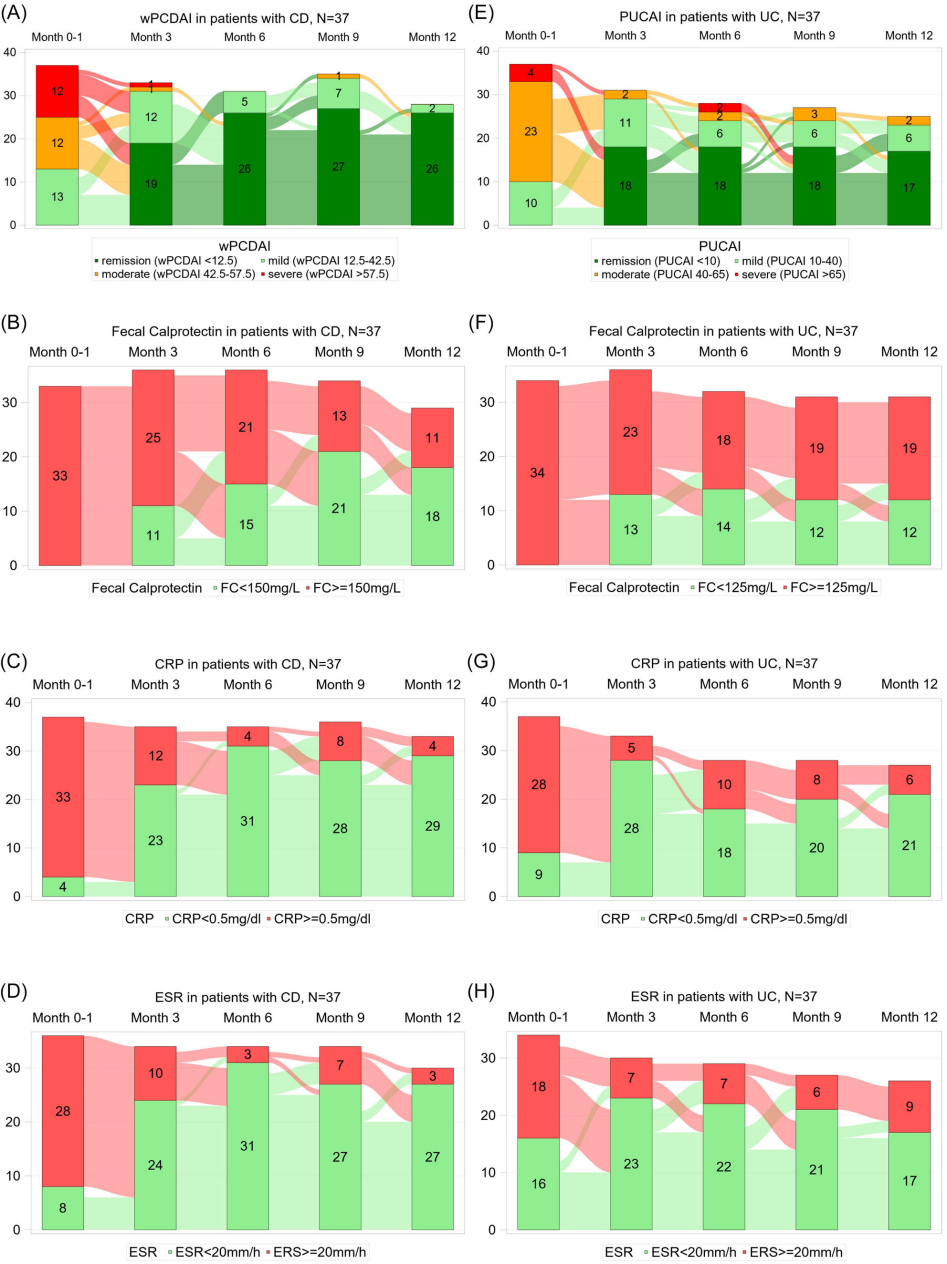
Development of wPCDAI or PUCAI and biomarkers in patients with CD (*N* = 37) and UC (*N* = 37) during the year after diagnosis. The Sankey diagrams illustrate the progression of wPCDAI (A) or PUCAI (E), FC (B, F), CRP (C, G) and ESR (D, H) in patients with CD and UC from diagnosis through follow‐up intervals of 1, 3, 6, and 12 months. CD, Crohn's disease; CRP, C‐reactive protein; ESR, erythrocyte sedimentation rate; FC, fecal calprotectin; PUCAI, Pediatric Ulcerative Colitis Activity Index; UC, Ulcerative colitis; wPCDAI, weighted Pediatric Crohn's Disease Activity Index.

**Figure 2:**
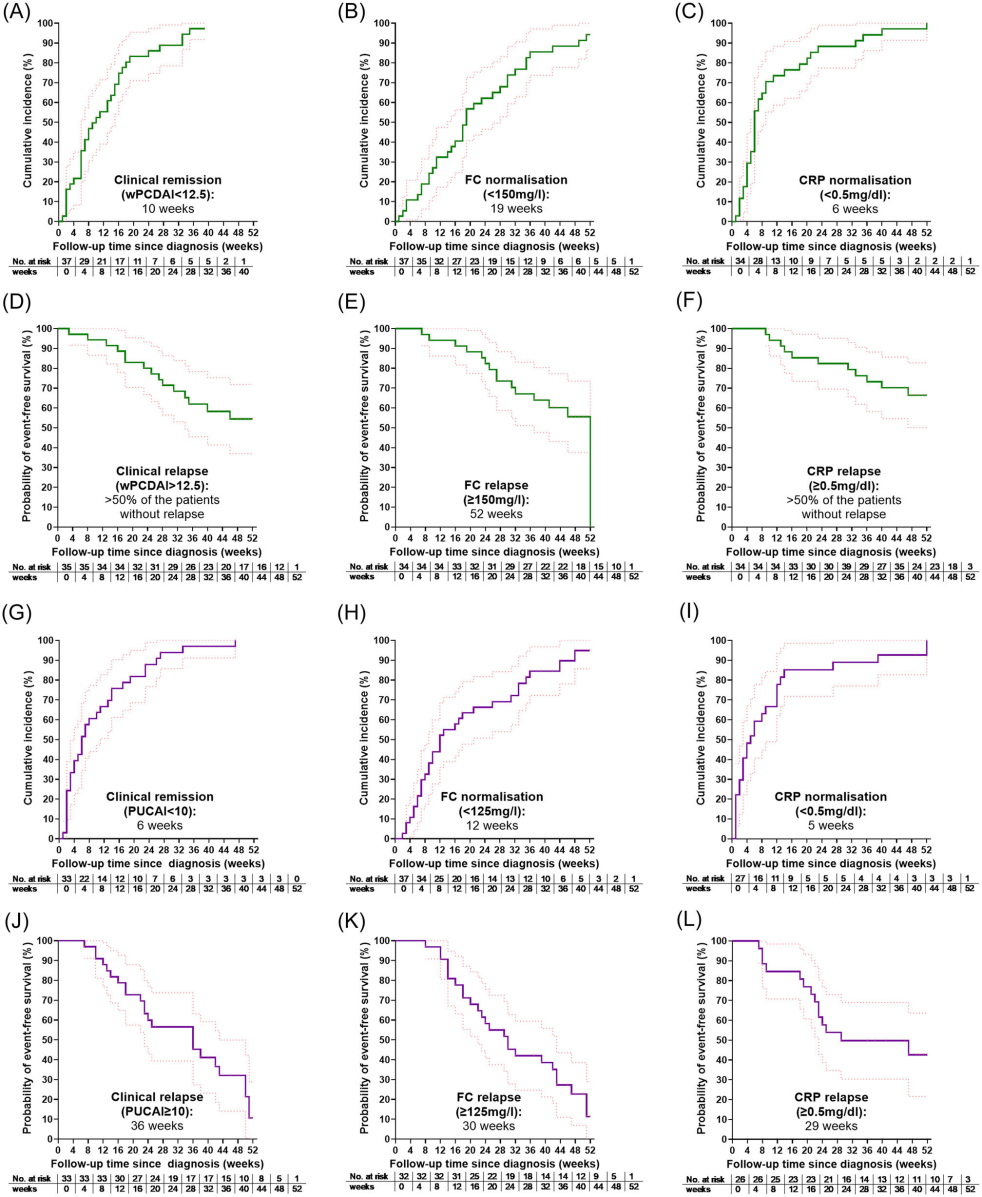
Clinical remission, treatment targets in biomarkers and relapse in patients with CD and UC. Kaplan–Meier curves demonstrate the time to clinical remission (A, G) and normalization of biomarkers FC (B, H) and CRP (C, I) in patients with CD (upper half in green) and UC (lower half in purple). The lower panels show time to clinical relapse after achieving the corresponding targets (D–F, J–L).CD, Crohn's disease; CRP, C‐reactive protein; ESR, erythrocyte sedimentation rate; FC, fecal calprotectin; PUCAI, Pediatric Ulcerative Colitis Activity Index; UC, Ulcerative colitis; wPCDAI, weighted Pediatric Crohn's Disease Activity Index.

The progression of wPCDAI or PUCAI in CD or UC patients during 1‐year follow‐up is illustrated in Figure 1A,E. Clinical remission was achieved in 35/37 (95%) CD patients and 33/37 (89%) UC patients during the 1‐year observation period (Table [Link], [Link]). CD patients reached remission after a median of 10 weeks (95% CI: 7–16; Figure 2A), whereas UC patients achieved remission faster, at a median of 6 weeks (95% CI: 4–14; Gray's test *p* = 0.3777; Figures 2G and S1A). Within 26 weeks, remission occurred in 31/37 (84%) CD and 30/37 (81%) UC patients (Table 2).

**Table 2:** Achievement of treatment targets within 26 weeks after diagnosis and endoscopy at follow‐up.

	CD			UC/IBD‐u		
Achievement of treatment targets within 26 weeks	Total (*N* = 37)	SES‐CD at follow‐up (*N* = 17)		Total (*N* = 37)	MES at follow‐up (*N* = 22)	
		SES‐CD < 3 or absence of ulcerations (*N* = 7)	SES‐CD ≥ 3 (*N* = 10)		MES = 0 (*N* = 6)	MES ≥ 1 (*N* = 16)
Clinical remission	31/37 (84%)	7/7 (100%)	10/10 (100%)	30/37 (81%)	6/6 (100%)	13/16 (81%)
FC normalization	24/37 (65%)	4/7 (57%)	10/10 (100%)	25/37 (68%)	5/6 (83%)	10/16 (63%)
CRP normalization	30/34^a^ (88%)	6/6^a^ (100%)	9/9^a^ (100%)	23/29^a^ (79%)	5/5^a^ (100%)	11/14^a^ (79%)
Clinical remission + FC **or** CRP normalization	30/37 (81%)	6/7 (86%)	10/10 (100%)	24/37 (65%)	5/6 (83%)	11/16 (69%)
Clinical remission + FC **and** CRP normalization	20/37 (54%)	4/7 (57%)	9/10 (90%)	16/37 (43%)	5/6 (83%)	6/16 (38%)

*Note*: The table lists the number of patients with CD and UC/IBD‐u who reached treatment targets—clinical remission, normalization of FC and CRP or their combination—within 26 weeks of diagnosis. It also shows how many patients with remission or active disease per endoscopic follow‐up achieved targets within 26 weeks. Endoscopic scores were available only for some patients. Abbreviations: CD, Crohn's disease; CRP, C‐reactive protein; FC, fecal calprotectin; IBD‐u, Inflammatory bowel disease‐unclassified; MES, Mayo endoscopic subscore; SES, simple endoscopic score; UC, ulcerative colitis.

^a^ No data on some patients at the 26‐week time point.

Within 52 weeks, 22/33 (67%) UC patients experienced clinical relapse, with 50% relapsing by 36 weeks (95% CI: 23–52; Figure 2J). In contrast, only 15/35 (43%) CD patients relapsed within 52 weeks (Figure 2D; Log‐rank *p* = 0.0334; Figure 1B).

### 3.4 Normalization of biomarkers

Sankey diagrams show the number of measurements and changes in biomarkers at diagnosis and throughout the 1‐year follow‐up in CD (Figure 1B–D) and UC (Figure 1F–H). Table 1 shows rates and median times to first FC normalization and FC relapse after remission. Median time to FC normalization was 19 weeks for CD and 12 weeks for UC (Figure 2B,H). Within 26 weeks, 24/37 (65%) CD and 25/37 (68%) UC patients achieved FC normalization (Gray's test *p* = 0.5531, Table 2, Figure S1). FC relapse occurred significantly earlier in UC than in CD (*p* = 0.0099, Figure S1). Median time to CRP normalization was 6 weeks in CD (95% CI: 5–9) and 5 weeks in UC (95% CI: 2–12; Figure 2C,I). Within 26 weeks, 30/34 (88%) CD and 23/29 (79%) UC patients achieved CRP normalization (Table 2). By 52 weeks, all CD patients and all but three UC patients reached CRP < 0.5 mg/dL. CRP relapse occurred in 11/34 (32%) CD and 14/26 (54%) UC patients (Log‐rank *p* = 0.0518, Figure S1).

### 3.5 Subgroup analysis before and after STRIDE‐II implementation

To assess the impact of the 2021 STRIDE publication and CD guideline update, patients treated before and after 2021 were compared (Figure S2). Clinical remission was achieved in 46/51 (90%) patients before 2021 and 22/23 (96%) after 2021, with median time to remission of 11 weeks (95% CI: 7–16) versus 6 weeks (95% CI: 6–10), respectively (Gray's test *p* = 0.1583). FC normalization occurred in 45/51 (88%) and 21/23 (91%) patients, with median times of 18 weeks (95% CI: 11–28) before and 16 weeks (95% CI: 10–32) after 2021 (*p* = 0.6948). ESR normalization was significantly faster after 2021 (16/16; median 6 weeks [95% CI: 4–15]) compared to before 2021 (38/40 [95%]; median 10 weeks [95% CI: 8–17]; *p* = 0.0189). Time‐to‐relapse after achieving targets did not differ significantly.

### 3.6 Endoscopic and histologic healing

At diagnosis, CD patients had a median SES‐CD of 10.5 (IQR: 6–15.5), and UC patients a median MES of 2 (IQR: 2–2). Follow‐up endoscopy within 24 months was available for 57% of CD and 65% of UC patients, at a median of 41 weeks (IQR: 33–54) and 52 weeks (IQR: 34–71), respectively. Among CD patients with SES‐CD data (*n* = 17), 7/17 (41%) achieved endoscopic healing (SES‐CD < 3 or absence of ulcerations); histologic healing was observed in 9/21 (43%). Among UC patients with MES data (*n* = 22), 13/22 (59%) achieved endoscopic healing (MES = 0) and 4/22 (18%) histologic healing (CD vs. UC *p* = 0.104; Table 1).

### 3.7 Resolution of growth impairment

Restoration of normal growth is a long‐term treatment target. Of 74 patients, 72 were included in the *z*‐score analysis (Figure S3; Tables S3 and S4). At baseline, one CD patient was excluded due to Down syndrome and one UC patient due to missing externally generated diagnostic data.

Growth impairment at diagnosis was present in 2/35 CD patients and in none with UC. Both CD patients achieved normal growth during follow‐up (Figure S3). In one patient, the height‐for‐age *z*‐score (haz) declined from 0.69 at baseline to –0.28 at last follow‐up (Δhaz = 0.97), exceeding the 0.75 threshold and indicating a significant reduction relative to diagnosis.

### 3.8 SDC within 26 weeks after diagnosis

To evaluate SDC in our pediatric cohort, we assessed the proportion of patients achieving or failing the treatment targets of clinical remission, FC normalization and CRP normalization at 26 and 52 weeks postdiagnosis.

Table 2 shows the numbers of CD and UC patients meeting targets at Week 26. Among 25 patients who did not achieve FC normalization by Week 26, 3 (12%) were lost to follow‐up. Therapeutic adjustments were made in 13/22 (59%) cases, including class switches in 6/13 (46%) CD and 6/9 (67%) UC patients, and dose escalation in one UC patient. Endoscopic outcomes at follow‐up are also presented. Table S2 shows target achievement within 52 weeks. Combined clinical remission and normalization of FC and CRP were observed in 20/37 (54%) CD patients at Week 26 and 29/37 (78%) at Week 52; in UC, 16/37 (43%) and 23/37 (62%), respectively.

No significant association was observed between target achievement at 26 or 52 weeks and follow‐up endoscopy outcomes. In CD, patients with SES‐CD < 3 or absence of ulcerations were paradoxically less likely to have met targets by Week 26, whereas in UC, achieving targets by Week 26 showed a trend toward better endoscopic outcomes (Table 2).

## 4 DISCUSSION

Based on updated STRIDE‐II recommendations, we analyzed outcomes of pediatric IBD patients during the first year post‐diagnosis in a real‐world setting, assessing time to clinical, fecal, and serum biomarker remission as well as relapse. Endoscopic targets were evaluated in a subset. Short‐term targets—clinical remission and CRP/ESR normalization—were achieved after a median of 5–10 weeks; FC normalization (intermediate target) after 12–19 weeks. By Week 26, 54% of CD and 43% of UC patients achieved combined targets of clinical remission and normalization of FC and CRP. Follow‐up endoscopy within 24 months showed endoscopic healing in 7/17 (41%) CD and 13/22 (59%) UC patients.

Our pediatric IBD cohort was representative regarding patient characteristics, IBD phenotype, inflammation markers, and growth impairment. Median age and gender distribution were comparable between CD and UC and aligned with epidemiological data. Growth failure, a major complication of pediatric IBD,^22^ has been reported in 15%–40% of childhood‐onset CD.^23^, ^24^ In our cohort, growth delay was observed less frequently in CD (2/37 patients), possibly due to short diagnostic latency in Munich. IBD phenotype, disease activity, and extraintestinal manifestations were similar to other pediatric studies.^25^, ^26^ As expected, systemic inflammation (elevated CRP and ESR) was more frequent in CD than in UC.^27^, ^28^

In our CD cohort, clinical remission was achieved after a median of 10 weeks, consistent with the STRIDE‐II‐recommended timeframe of 4–17 weeks, depending on therapy. Whether earlier assessments would have shortened time‐to‐remission remains uncertain. CRP and ESR normalized largely within expected ranges.^17^ Median time to CRP normalization was 6 weeks, aligning with the anticipated 5–15 weeks.^17^ In new onset pediatric CD, CRP has been shown to normalize within 12 weeks with steroid or EEN treatment, and among those in steroid‐free remission in Week 12, normal CRP predicted 1‐year sustained remission.^29^ FC, a widely used IBD biomarker with 82% sensitivity and 72% specificity for endoscopic activity in CD,^30^ is recommended in STRIDE‐II as a prognostic treatment target, with a suggested cutoff of 150 mg/l.^17^, ^31^ In our cohort, FC normalization occurred at a median of 19 weeks, later than reported in adult studies, possibly reflecting the more extensive disease phenotype typically observed in pediatric IBD.^6^

UC patients achieved clinical remission after a median of 6 weeks, aligning with STRIDE‐II recommendations.^12^ Since clinical symptoms closely reflect endoscopic inflammation in UC, normalization of stool frequency and absence of rectal bleeding are key treatment goals.^17^ While CRP and ESR correlate only modestly with endoscopic activity, FC is a more reliable marker of both clinical and endoscopic disease.^32^, ^33^ In our cohort, the intermediate target FC normalization occurred after a median of 12 weeks. This is comparable to a previous study, in which median time‐to‐reach target FC <250mg/l was 11 weeks in pediatric patients with UC.^31^ FC has been demonstrated to predict clinical and endoscopic relapse in both adult and pediatric IBD.^34^, ^35^, ^36^ In children, FC < 300 µg/g moderately predicts mucosal healing, while a lower cutoff <100 µg/g may better indicate transmural healing. ^37^

In the IBD‐PODCAST study by D'Amico et al., SDC occured in 52.2% of CD and 44.3% of UC patients (≥19 years, *n* = 2185, 10 countries).^21^ Similar rates were observed in the United Kingdom (CD: 52.4%, UC: 45.3%)^38^ and Italian cohorts (CD: 53.4%, UC: 49.0%).^39^ Contributing factors included poor quality of life, extraintestinal manifestations, steroid overuse, ongoing inflammation, and absent endoscopic remission.^21^ In our pediatric cohort, only clinical activity and biomarker normalization were evaluated; quality of life and standardized follow‐up endoscopies were not assessed. As a key difference, our patients were newly diagnosed, with short disease duration. At 26 weeks, 7/37 (19%) CD and 13/37 (35%) UC patients had not achieved clinical remission plus FC or CRP normalization. At 52 weeks, 2/37 (5%) CD and 6/37 (16%) UC patients had not yet met these targets, reflecting higher rates of SDC in UC.

Follow‐up endoscopy within 24 months was performed in 57% of CD (SES‐CD available for 18) and 65% of UC patients (MES for 22) indicating that endoscopic healing was not assessed in approximately one‐third of patients. This may have several explanations. In pediatric practice, endoscopy is often avoided because it typically requires deep sedation or general anesthesia and imposes substantial burden related to bowel preparation, IV access, fasting, hospital visits, and procedure‐related anxiety; consequently, it is usually reserved for children with significant symptom burden.^40^ Non‐invasive tools such as the Mucosal Inflammation Noninvasive Index (MINI‐index) and bowel ultrasound therefore play an important role in assessing mucosal healing.^41^, ^42^ To better address pediatric needs, future studies should evaluate the applicability of adult endoscopy‐timing recommendations in children.

In our cohort, early anti‐TNF use was significantly more common in CD than UC, possibly reflecting the established top‐down strategy in CD. Predictors of poor outcome enable early selection of appropriate induction therapy, including initial anti‐TNF use in high‐risk CD patients.^16^ In UC, however, validated predictors of poor outcome are lacking, and a step‐up approach starting with 5‐ASA and steroids remains standard, except in steroid‐refractory acute severe colitis.

UC patients showed earlier and more frequent relapses, underscoring limitations of conventional therapy. This supports STRIDE‐II recommendations for individualized targets, including clinical and endoscopic remission.^17^ Future studies should assess whether early biologics confer similar benefits in UC as in CD. Non‐invasive tools like bowel ultrasound may also complement endoscopy, offering a patient‐friendly approach to disease monitoring.^17^

This single‐center study is limited by its relatively small sample size and the constraints of real‐world retrospective design. Non‐standardized visit schedules may have introduced inaccuracies in time‐to‐target analyses, as treatment goals could have been reached earlier than documented. Assessment of endoscopic healing was limited by variable timing of follow‐up procedures. Quality of life and disability could not be evaluated because standardized instruments such as IMPACT‐III were not applied. Moreover, thresholds for endoscopic remission or response remain unvalidated and require further investigation.^17^ Histologic healing in UC and transmural healing in CD are not formal STRIDE‐II targets, as their prognostic value and the therapeutic burden of achieving them remain unclear. Nonetheless, both are emerging as relevant adjunctive endpoints.^17^

## 5 CONCLUSION

This study demonstrates that short‐ and intermediate‐term targets by STRIDE‐II, were achieved earlier in patients with UC than in patients with CD. Clinical relapse within the first year post‐diagnosis occurred earlier and more frequently in UC than in CD patients. The application of STRIDE‐II criteria, incorporating endoscopic targets alongside histological remission, has proven feasible and valuable for therapy monitoring in real‐world settings. The lower relapse rates observed in CD further support the efficacy of a top‐down therapeutic approach. Nevertheless, the substantial proportion of patients with SDC and the more extensive disease phenotype in children highlight the need for continuous monitoring in pediatric IBD, including non‐invasive and practical modalities such as ultrasound for repeated assessment of disease activity and treatment response. Despite this, regular monitoring remains a considerable challenge in clinical practice, and addressing these limitations is essential to optimize long‐term disease management.

## CONFLICT OF INTEREST STATEMENT

Hannes Hoelz received speaker's fee from Nutricia/Danone and travel expenses from IPSEN outside of the submitted work. Tobias Schwerd received speaker's fees from MSD and Nutricia, and travel support from Nestlé Nutrition outside of the submitted work. Sibylle Koletzko received fees as member of advisory boards or as speaker from AbbVie, AstraZeneca, Danone, Janssen, Mead Johnson, Nestlé Nutrition, Pfizer, Sanofi, Takeda outside of the submitted work. The remaining authors declare no conflicts of interest. This work received no financial support from third parties (e.g., pharmaceuticals, industry, organizations).

## Supporting information

Supplemental Figure S1_final.

Supplemental Figure S2_final.

Supplemental Figure S3.

Supplemental Table S1.

Supplemental Table S2.

Supplemental Table S3.

Supplemental Table S4.
