# Supplementary material for: Real‐world data on STRIDE‐II treatment targets in a pediatric cohort with inflammatory bowel disease
Source: J Pediatr Gastroenterol Nutr. 2026 Jan 18;82(4):1006–18. doi: 10.1002/jpn3.70345 (PMC13050806; doi:10.1002/jpn3.70345)
Supplement: Supplementary file 1 — Supplemental Figure S1_final. [file JPN3-82-1006-s003.docx]

| **Disease activity index (wPCDAI or PUCAI), comparison between CD and UC** | |
| --- | --- |
| 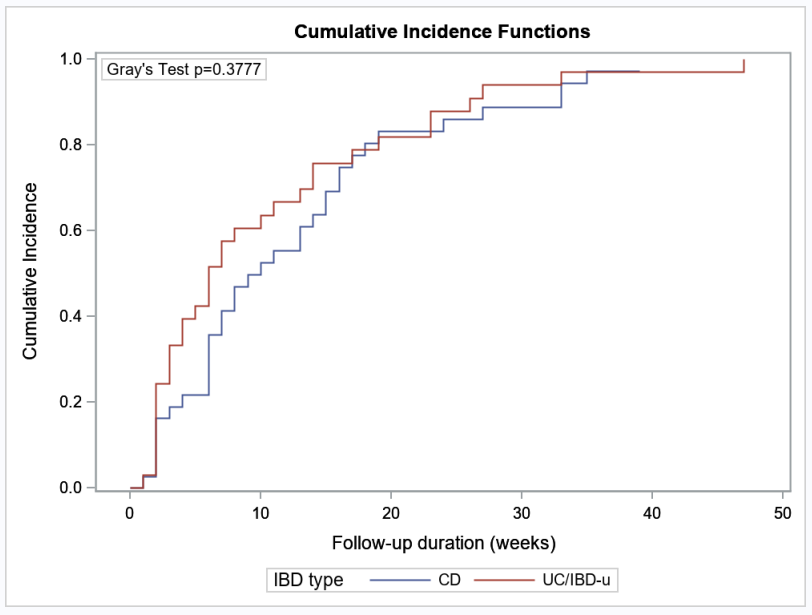**a)Time-to-clinical remission**(CD: wPCDAI <12.5; UC: PUCAI <10)  Cumulative number of patients reached clinical remission     \| **F/u week** \| **0** \| **4** \| **6** \| **12** \| **18** \| **24** \| **26** \| **30** \| **36** \| **42** \| **48** \| **52** \| \| --- \| --- \| --- \| --- \| --- \| --- \| --- \| --- \| --- \| --- \| --- \| --- \| --- \| \| N (CD) \| 0 \| 8 \| 13 \| 20 \| 29 \| 31 \| 31 \| 32 \| 35 \| 35 \| . \| . \| \| N (UC) \| 0 \| 13 \| 17 \| 22 \| 26 \| 29 \| 30 \| 31 \| 32 \| 32 \| 33 \| . \|     Gray’s test *p = 0.377* | **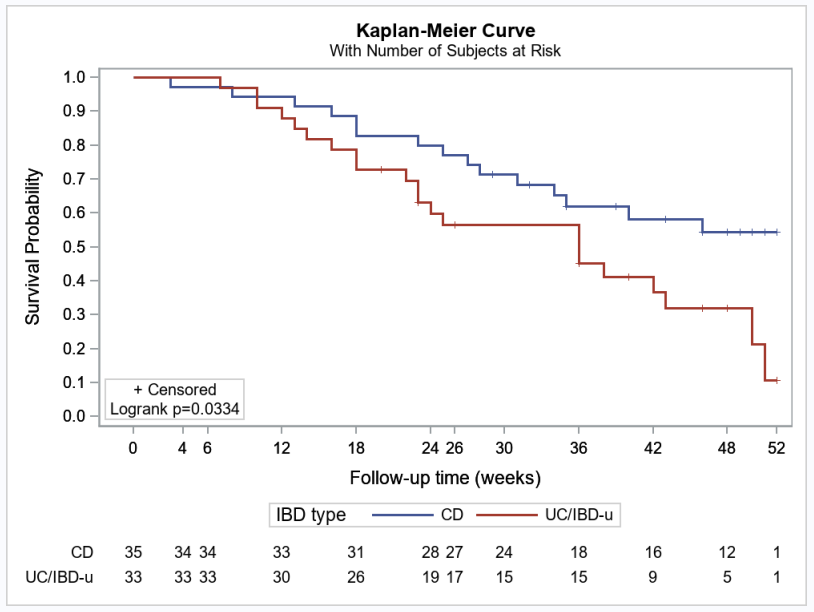b) Time-to-clinical relapse**(CD: wPCDAI >12.5, UC: PUCAI ≥10)  Number of patients at risk     \| **F/u week** \| **0** \| **4** \| **6** \| **12** \| **18** \| **24** \| **26** \| **30** \| **36** \| **42** \| **48** \| **52** \| \| --- \| --- \| --- \| --- \| --- \| --- \| --- \| --- \| --- \| --- \| --- \| --- \| --- \| \| N (CD) \| 35 \| 34 \| 34 \| 33 \| 31 \| 28 \| 27 \| 24 \| 18 \| 16 \| 12 \| 1 \| \| N (UC) \| 33 \| 33 \| 33 \| 30 \| 26 \| 19 \| 17 \| 15 \| 15 \| 9 \| 5 \| 1 \|     Log-Rank ***p = 0.0334***  Peto-Peto *p = 0.0641*  Tarone-Ware *p = 0.0646* |
| **Fecal calprotectin, comparison between CD and UC** | |
| 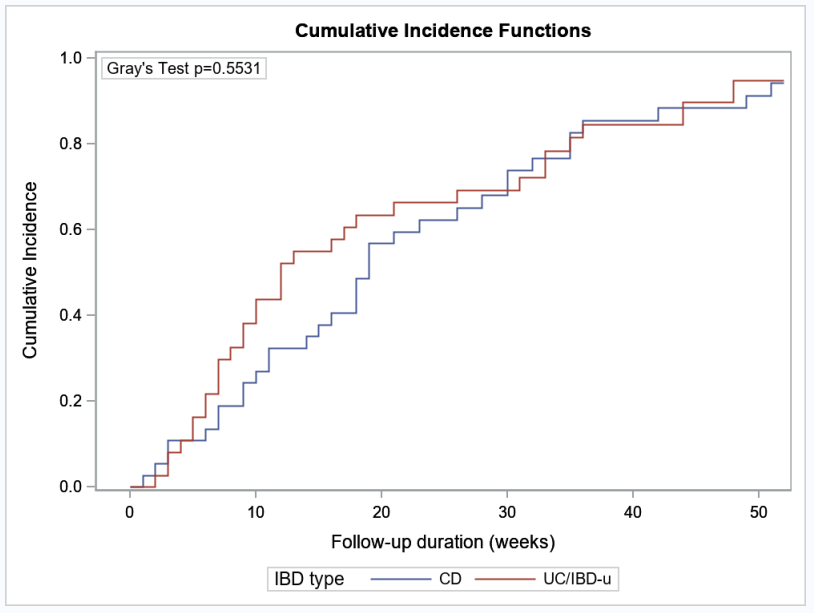**c)** **Time-to-FC normalization**(CD: <150mg/l; UC: <125mg/l)  Cumulative number of patients reached clinical remission     \| **F/u week** \| **0** \| **4** \| **6** \| **12** \| **18** \| **24** \| **26** \| **30** \| **36** \| **42** \| **48** \| **52** \| \| --- \| --- \| --- \| --- \| --- \| --- \| --- \| --- \| --- \| --- \| --- \| --- \| --- \| \| N (CD) \| 0 \| 4 \| 5 \| 12 \| 18 \| 23 \| 24 \| 27 \| 31 \| 32 \| 32 \| 34 \| \| N (UC) \| 0 \| 4 \| 8 \| 19 \| 23 \| 24 \| 25 \| 25 \| 30 \| 30 \| 32 \| 32 \|     Gray’s test *p = 0.5531* | 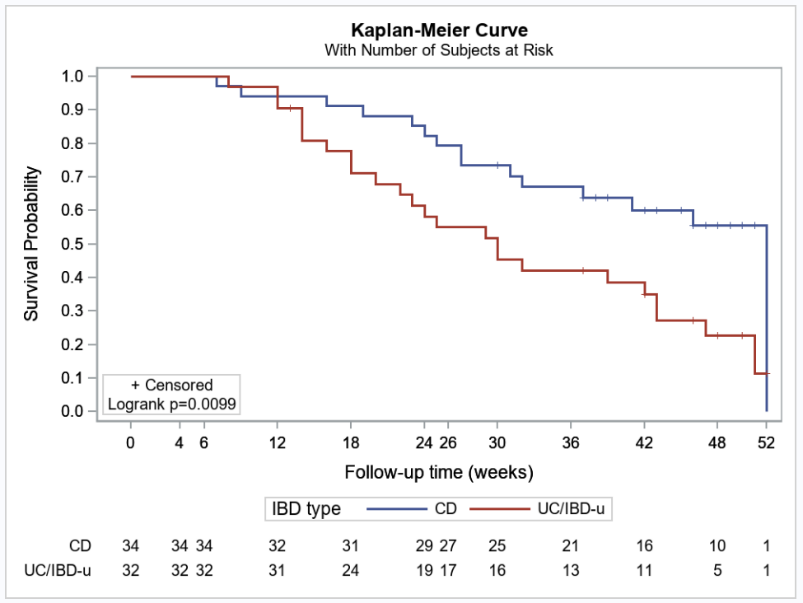**d)** **FC relapse**(CD: ≥150mg/l, UC: ≥125mg/l)  Number of patients at risk     \| **F/u week** \| **0** \| **4** \| **6** \| **12** \| **18** \| **24** \| **26** \| **30** \| **36** \| **42** \| **48** \| **52** \| \| --- \| --- \| --- \| --- \| --- \| --- \| --- \| --- \| --- \| --- \| --- \| --- \| --- \| \| N (CD) \| 34 \| 34 \| 34 \| 32 \| 31 \| 29 \| 27 \| 25 \| 21 \| 16 \| 10 \| 1 \| \| N (UC) \| 32 \| 32 \| 32 \| 31 \| 24 \| 19 \| 17 \| 16 \| 13 \| 11 \| 5 \| 1 \|     Log-Rank ***p = 0.0099***  Peto-Peto ***p = 0.0114***  Tarone-Ware ***p = 0.0103*** |
| **C-reactive protein, comparison between CD and UC** | |
| Analysis of time-to-CRP normalization is not applicable, because only 3 patients with UC did not reach CRP <0.5mg/dl, while all patients with CD reached CRP <0.5mg/dl. | **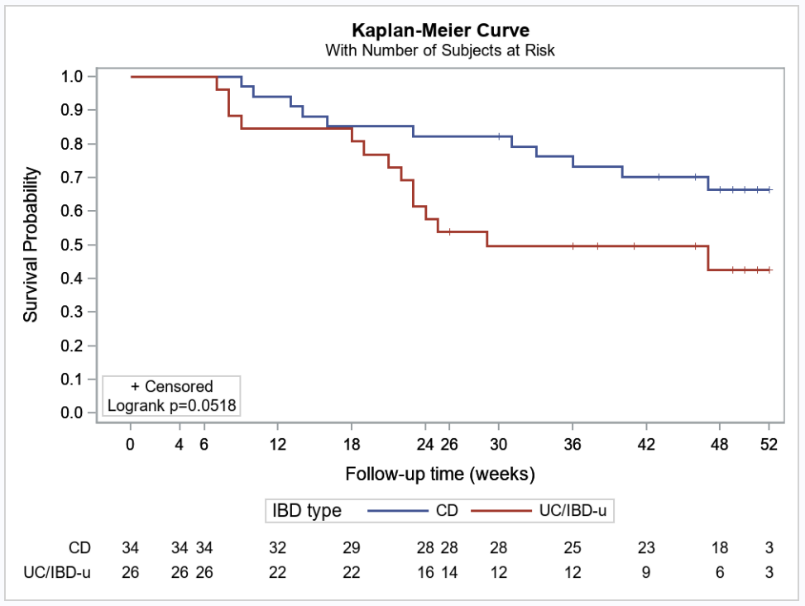e) CRP relapse**(CD & UC: ≥0.5mg/dl)  Number of patients at risk     \| **F/u week** \| **0** \| **4** \| **6** \| **12** \| **18** \| **24** \| **26** \| **30** \| **36** \| **42** \| **48** \| **52** \| \| --- \| --- \| --- \| --- \| --- \| --- \| --- \| --- \| --- \| --- \| --- \| --- \| --- \| \| N (CD) \| 34 \| 34 \| 34 \| 32 \| 29 \| 28 \| 28 \| 28 \| 25 \| 23 \| 18 \| 3 \| \| N (UC) \| 26 \| 26 \| 26 \| 22 \| 22 \| 16 \| 14 \| 12 \| 12 \| 9 \| 6 \| 3 \|     Log-Rank *p = 0.0518*  Peto-Peto ***p = 0.0482***  Tarone-Ware ***p = 0.0477*** |
| **Erythrocyte Sedimentation Rate, comparison between CD and UC** | |
| **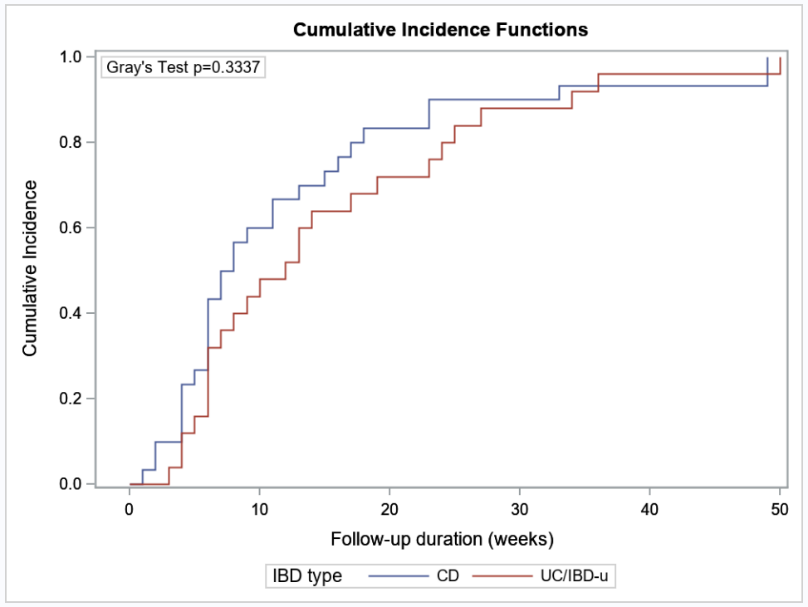f) Time-to-ESR normalization**(CD & UC: ESR <20mm/h)    Cumulative number of patients reached clinical remission     \| **F/u week** \| **0** \| **4** \| **6** \| **12** \| **18** \| **24** \| **26** \| **30** \| **36** \| **42** \| **48** \| **52** \| \| --- \| --- \| --- \| --- \| --- \| --- \| --- \| --- \| --- \| --- \| --- \| --- \| --- \| \| N (CD) \| 0 \| 7 \| 13 \| 20 \| 25 \| 27 \| 27 \| 27 \| 28 \| 28 \| 28 \| 29 \| \| N (UC) \| 0 \| 3 \| 8 \| 13 \| 17 \| 20 \| 21 \| 22 \| 24 \| 24 \| 24 \| 25 \|     Gray’s test *p = 0.3337* | **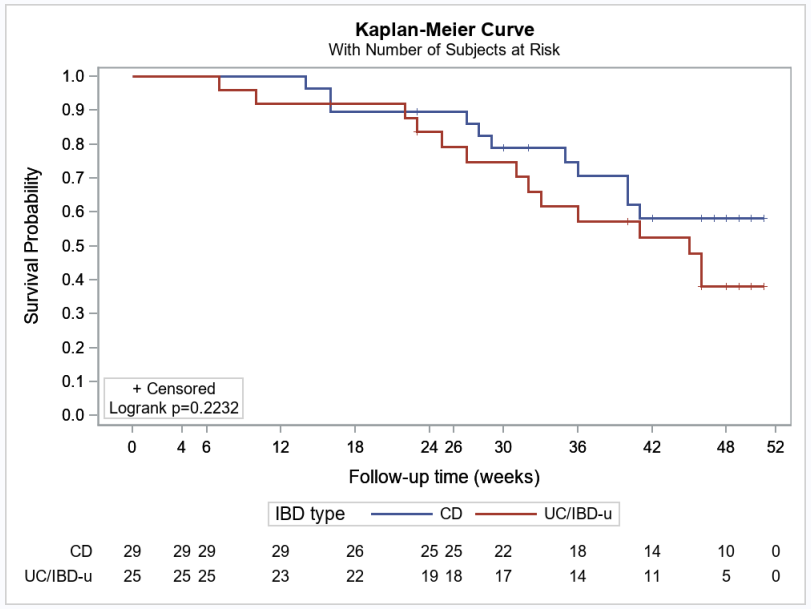g) ESR relapse**(CD & UC: ≥20mm/h)    Number of patients at risk     \| **F/u week** \| **0** \| **4** \| **6** \| **12** \| **18** \| **24** \| **26** \| **30** \| **36** \| **42** \| **48** \| **52** \| \| --- \| --- \| --- \| --- \| --- \| --- \| --- \| --- \| --- \| --- \| --- \| --- \| --- \| \| N (CD) \| 29 \| 29 \| 29 \| 29 \| 26 \| 25 \| 25 \| 22 \| 18 \| 14 \| 10 \| 0 \| \| N (UC) \| 25 \| 25 \| 25 \| 23 \| 22 \| 19 \| 18 \| 17 \| 14 \| 11 \| 5 \| 0 \|     Log-Rank *p = 0.2232*  Peto-Peto *p = 0.2546*  Tarone-Ware *p = 0.2537* |
| **Supplementary Figure 1: Comparison of outcome parameters in patients with Crohn’s disease (CD) and Ulcerative colitis (UC): Time to clinical remission, normalization of biomarkers, and time to clinical relapseafter achieving the respective treatment targets.**  Kaplan-Meier curves on the left side demonstrate the time to clinical remission **(a)** and normalization of biomarkers fecal calprotectin **(c)**, and Erythrocyte Sedimentation Rate **(f)** in patients with Crohn's disease (CD, blue) and Ulcerative colitis or Inflammatory Bowel Disease-unclassified (UC/IBD-u, red). The panels on the right side show the time to clinical relapse after achieving the respective treatment targets **(b, d, e, g)**. Clinical remission was defined as wPCDAI <12.5 points for CD patients or PUCAI <10 points for UC patients. Clinical relapse was defined as an increase in wPCDAI to ≥12.5 points or PUCAI to ≥10 points after having achieved remission. Biomarker normalization was defined as FC <150mg/l for CD and <125 mg/l for UC, ESR <20mm/h, and CRP <0.5 mg/dL for both conditions.  In the time-to-reach treatment target analyses p-value was obtained using Gray’s test to compare the cumulative incidence functions between groups. To compare survival distributions between groups -value was obtained using the log-rank test. To account for potential violations of the proportional hazard assumption, additional p-values from Tarone–Ware and Peto–Peto tests were reported.    Abbreviations: CD = Crohn‘s disease, CRP = C-reactive protein, FC = fecal calprotectin, IBD-u = Inflammatory Bowel Disease-unclassified, PUCAI = Pediatric Ulcerative Colitis Activity Index, UC = Ulcerative colitis, wPCDAI = weighted Pediatric Crohn´s Disease Activity Index. | |
