# Supplementary material for: Real‐world data on STRIDE‐II treatment targets in a pediatric cohort with inflammatory bowel disease
Source: J Pediatr Gastroenterol Nutr. 2026 Jan 18;82(4):1006–18. doi: 10.1002/jpn3.70345 (PMC13050806; doi:10.1002/jpn3.70345)
Supplement: Supplementary file 3 — Supplemental Figure S3. [file JPN3-82-1006-s006.docx]

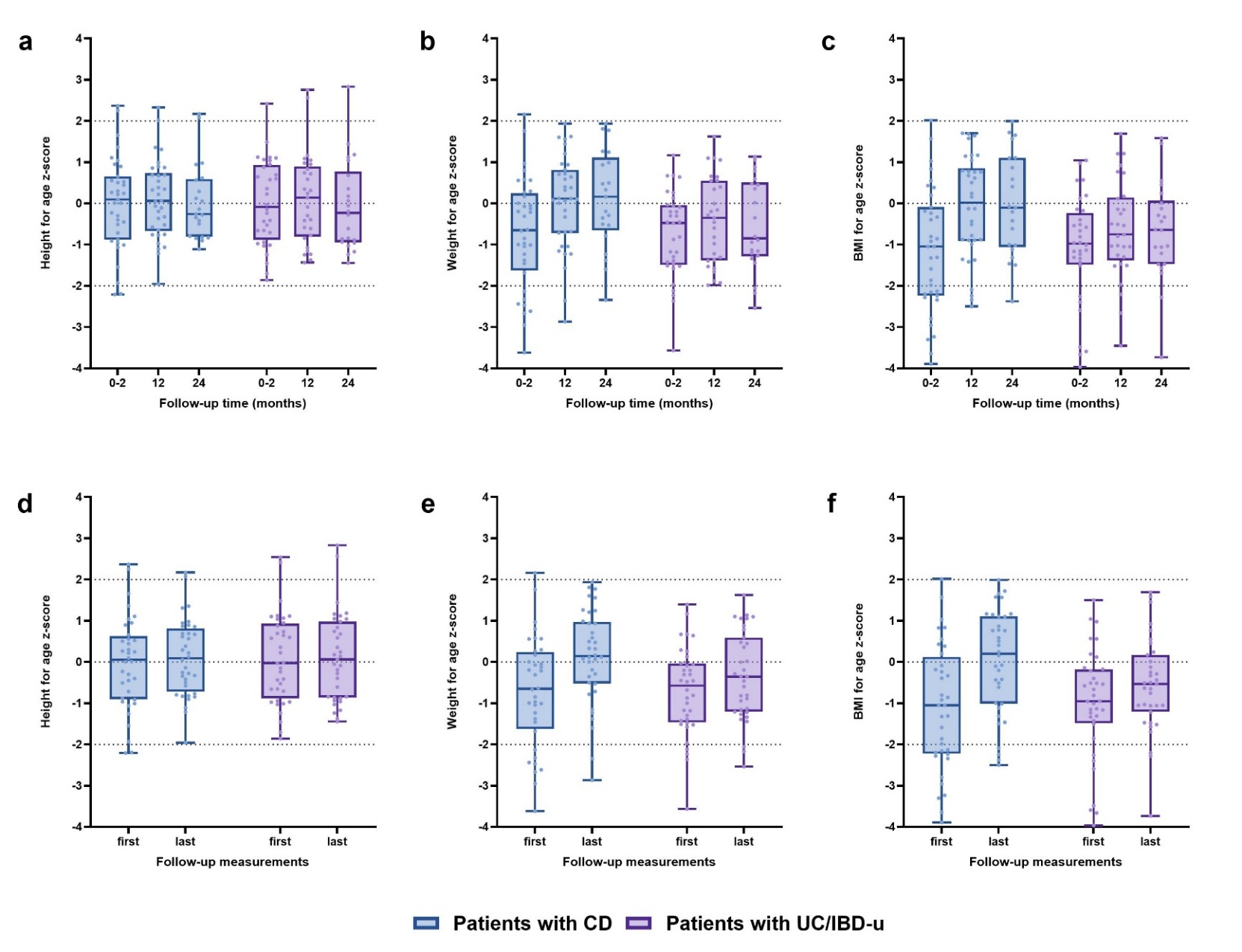


**Supplemental Figure S3: Growth impairment and development in patients with** **Crohn´s disease (CD) and Ulcerative colitis (UC) / Inflammatory bowel disease-unclassified (IBD-u).**

Height-for-age z-scores (haz), weight-for-age z-scores (waz), and BMI z-scores (bmiz) for availiable measurements obtained at 0-2, within a 12- and 24-month follow-up period **(a-c)**, as well as the initial and last measurement during follow-up period **(d-f),** are shown.
