# Supplementary material for: Real‐world data on STRIDE‐II treatment targets in a pediatric cohort with inflammatory bowel disease
Source: J Pediatr Gastroenterol Nutr. 2026 Jan 18;82(4):1006–18. doi: 10.1002/jpn3.70345 (PMC13050806; doi:10.1002/jpn3.70345)
Supplement: Supplementary file 4 — Supplemental Table S1. [file JPN3-82-1006-s007.docx]

**Supplemental Table S1: Number of measurements in pediatric patients with Crohn´s disease (CD) and Ulcerative colitis (UC)**

| Number of measurements within 52 weeks since diagnosis, median (IQR) | Crohn´s disease (CD)  (N = 37) | Ulcerative colitis (UC) / Inflammatory bowel disease-unclassified (IBD-u)  (N = 37) |
| --- | --- | --- |
| wPCDAI | 9 (7-11)  min-max: 2-14 | n.a. |
| PUCAI | n.a. | 8 (5-11)  min-max: 1-20 |
| FC | 9 (7-10)  min-max: 4-17 | 9 (6-12)  min-max: 2-17 |
| CRP | 10 (9-12)  min-max: 4-17 | 9 (6-13)  min-max: 1-19 |
| ESR | 9 (7-11)  min-max: 2-13 | 7 (5-11)  min-max: 1-18 |

Table showing the number of measurements for wPCDAI, PUCAI, FC, CRP and ESR recorded for each patient within 52 weeks after diagnosis. Median number with IQR and minimum and maximum number are given.

Abbreviation: CRP = C-reactive protein, ESR = erythrocyte sedimentation rate, FC = Fecal Calprotectin, IQR = interquartile range (25th and 75th percentile), n.a.: = not applicable, PUCAI = Pediatric Ulcerative Colitis Activity Index, wPCDAI = weighted Pediatric Crohn´s Disease Activity Index.
