# Supplementary material for: Real‐world data on STRIDE‐II treatment targets in a pediatric cohort with inflammatory bowel disease
Source: J Pediatr Gastroenterol Nutr. 2026 Jan 18;82(4):1006–18. doi: 10.1002/jpn3.70345 (PMC13050806; doi:10.1002/jpn3.70345)
Supplement: Supplementary file 5 — Supplemental Table S2. [file JPN3-82-1006-s004.docx]

**Supplemental Table S2: Achievement of treatment targets within 52 weeks after diagnosis and endoscopy at follow-up**

|  | Crohn’s disease (CD) | | | Ulcerative colitis (UC) / Inflammatory bowel disease-unclassified (IBD-u) | | |
| --- | --- | --- | --- | --- | --- | --- |
| Achievement of treatment targets within 52 weeks | Total  (N = 37) | SES-CD at follow-up  (N = 17) | | Total  (N = 37) | MES at follow-up  (N = 22) | |
|  |  | SES-CD <3 or absence of ulcerations  (N = 7) | SES-CD ≥3  (N = 10) |  | MES =0  (N = 13) | MES ≥1  (N = 9) |
| Clinical remission | 35/37 (95%) | 7/7  (100%) | 10/10 (100%) | 33/37 (89%) | 13/13  (100%) | 9/9  (100%) |
| FC normalization | 34/37 (92%) | 7/7  (100%) | 10/10 (100%) | 32/37 (86%) | 13/13  (100%) | 9/9  (100%) |
| CRP normalization | 34/34^+^ (100%) | 6/6^+^  (100%) | 9/9^+^  (100%) | 26/29^+^ (90%) | 11/11^+^  (100%) | 8/8^+^  (100%) |
| Clinical remission + FC **or** CRP normalization | 35/37 (95%) | 7/7  (100%) | 10/10  (100%) | 31/37 (84%) | 13/13  (100%) | 9/9  (100%) |
| Clinical remission + FC **and** CRP normalization | 29/37 (78%) | 6/7  (86%) | 9/10  (90%) | 23/37 (62%) | 10/13  (77%) | 9/9  (89%) |

^+^ no data on some patients at the 26-week time point

The table lists the number of patients with CD and UC/IBD-u who reached treatment targets – clinical remission, normalization of FC and CRP or their combination – within 52 weeks of diagnosis. It also shows how many patients with remission or active disease per endoscopic follow-up achieved targets within 52 weeks. Endoscopic scores were available only for some patients.

Abbreviations: CRP = C-reactive Protein, FC = Fecal Calprotectin, MES = Mayo endoscopic subscore, SES-CD = Simple Endoscopic Score.
