# Supplementary material for: Real‐world data on STRIDE‐II treatment targets in a pediatric cohort with inflammatory bowel disease
Source: J Pediatr Gastroenterol Nutr. 2026 Jan 18;82(4):1006–18. doi: 10.1002/jpn3.70345 (PMC13050806; doi:10.1002/jpn3.70345)
Supplement: Supplementary file 6 — Supplemental Table S3. [file JPN3-82-1006-s005.docx]

**Supplemental Table S3: Evaluation of growth impairment within 12 months with height-for-age z-scores (haz)**

|  | Crohn’s disease (CD) (N=34) | Ulcerative colitis (UC) / Inflammatory bowel disease-unclassified (IBD-u) (N=29) | Total (N=63) | P-value |
| --- | --- | --- | --- | --- |
| **Time difference between the first and last measurement within 12 months (±2 months) since diagnosis** | | | | 0.09^1^ |
| N (Missing) | 34 (0) | 29 (0) | 63 (0) |  |
| Range | 6.00, 14.00 | 3.00, 14.00 | 3.00, 14.00 |  |
| Mean (SD) | 12.15 (2.30) | 10.72 (3.37) | 11.49 (2.91) |  |
| Median (IQR) | 13.00 (11.00, 14.00) | 12.00 (8.00, 13.00) | 12.00 (11.00, 14.00) |  |
| **Difference of the last haz (within 12 month period ) and first haz (at baseline)** | | | | 0.44^1^ |
| N (Missing) | 34 (0) | 29 (0) | 63 (0) |  |
| Range | -1.55, 0.36 | -0.71, 0.71 | -1.55, 0.71 |  |
| Mean (SD) | -0.08 (0.38) | 0.00 (0.33) | -0.04 (0.36) |  |
| Median (IQR) | 0.01 (-0.22, 0.15) | 0.07 (-0.21, 0.19) | 0.02 (-0.22, 0.18) |  |
| **height-for-age z-score (haz), last measurement within 12 months (±2 months) since diagnosis was analyzed** | | | | 0.86^1^ |
| N (Missing) | 34 (0) | 29 (0) | 63 (0) |  |
| Range | -1.96, 2.32 | -1.43, 2.75 | -1.96, 2.75 |  |
| Mean (SD) | 0.09 (0.95) | 0.10 (1.10) | 0.10 (1.01) |  |
| Median (IQR) | 0.06 (-0.65, 0.69) | 0.14 (-0.77, 0.84) | 0.07 (-0.75, 0.84) |  |
| ^1^Wilcoxon rank sum p-value | | | | |
