# Supplementary material for: Real‐world data on STRIDE‐II treatment targets in a pediatric cohort with inflammatory bowel disease
Source: J Pediatr Gastroenterol Nutr. 2026 Jan 18;82(4):1006–18. doi: 10.1002/jpn3.70345 (PMC13050806; doi:10.1002/jpn3.70345)
Supplement: Supplementary file 7 — Supplemental Table S4. [file JPN3-82-1006-s001.docx]

**Supplemental Table S4: Evaluation of growth impairment within 24 months with height-for-age z-scores (haz)**

|  | Crohn´s disease (CD) (N=21) | Ulcerative colitis (UC) / Inflammatory bowel disease-unclassified (IBD-u) (N=22) | Total (N=43) | P-value |
| --- | --- | --- | --- | --- |
| **Time difference between the first and last measurement within 24 months (±2 months) since diagnosis** | | | | 0.96^1^ |
| N (Missing) | 21 (0) | 22 (0) | 43 (0) |  |
| Range | 15.00, 26.00 | 17.00, 26.00 | 15.00, 26.00 |  |
| Mean (SD) | 22.52 (3.82) | 22.82 (3.39) | 22.67 (3.56) |  |
| Median (IQR) | 24.00 (19.00, 26.00) | 25.00 (19.00, 25.00) | 25.00 (19.00, 26.00) |  |
| **Difference of the last haz (within 24 month period ) and first haz (at baseline)** | | | | 0.43^1^ |
| N (Missing) | 21 (0) | 22 (0) | 43 (0) |  |
| Range | -1.70, 0.33 | -1.12, 0.97 | -1.70, 0.97 |  |
| Mean (SD) | -0.17 (0.47) | 0.01 (0.48) | -0.08 (0.48) |  |
| Median (IQR) | -0.06 (-0.38, 0.18) | -0.09 (-0.29, 0.30) | -0.08 (-0.29, 0.20) |  |
| **height-for-age z-score (haz), last measurement within 24 months (±2 months) since diagnosis was analyzed** | | | | 0.52^1^ |
| N (Missing) | 21 (0) | 22 (0) | 43 (0) |  |
| Range | -1.11, 2.17 | -1.45, 2.83 | -1.45, 2.83 |  |
| Mean (SD) | 0.03 (0.93) | -0.07 (1.09) | -0.02 (1.00) |  |
| Median (IQR) | -0.26 (-0.80, 0.57) | -0.23 (-0.93, 0.68) | -0.26 (-0.85, 0.62) |  |
| ^1^Wilcoxon rank sum p-value | | | | |
